# Reprocessable Epoxy–Anhydride Resin Enabled by a Thermally Stable Liquid Transesterification Catalyst

**DOI:** 10.3390/polym16223216

**Published:** 2024-11-20

**Authors:** Huan Liang, Wendi Tian, Hongtu Xu, Yuzhen Ge, Yang Yang, Enjian He, Zhijun Yang, Yixuan Wang, Shuhan Zhang, Guoli Wang, Qiulin Chen, Yen Wei, Yan Ji

**Affiliations:** 1 Key Laboratory of Bioorganic Phosphorus Chemistry & Chemical Biology, Department of Chemistry, Tsinghua University, Beijing 100084, China; liangh1305@163.com (H.L.); xuht0929@163.com (H.X.); samaichi030156@163.com (Y.G.); hej21@mails.tsinghua.edu.cn (E.H.); yang-zj22@mails.tsinghua.edu.cn (Z.Y.); yx-wang23@mails.tsinghua.edu.cn (Y.W.); sh-zhang24@mails.tsinghua.edu.cn (S.Z.); 2 School of Materials Science and Engineering, University of Science and Technology Beijing, Beijing 100083, China; wendi0820hjj@163.com; 3 Institute of Nuclear and New Energy Technology, Tsinghua University, Beijing 100084, China; yangyang86@tsinghua.edu.cn; 4 Electric Power Research Institute, China Southern Power Grid Co., Ltd., Guangzhou 510623, China; wanggl@csg.cn (G.W.); chenql5@csg.cn (Q.C.)

**Keywords:** epoxy, transesterification, vitrimer, dynamic covalent bond, reprocessability

## Abstract

Introducing dynamic ester bonds into epoxy–anhydride resins enhances the reprocessability of the crosslinked network, facilitated by various types of transesterification catalysts. However, existing catalysts, such as metal salts and organic molecules, often struggle with dispersion, volatility, or structural instability issues. Here, we propose to solve such problems by incorporating a liquid-state, thermally stable transesterification catalyst into epoxy resins. This catalyst, an imidazole derivative, can be uniformly dispersed in the epoxy resin at room temperature. In addition, it shows high-temperature structural stability above at least 200 °C as the synergistic effects of the electron-withdrawing group and steric bulk can be leveraged. It can also effectively promote transesterification at elevated temperatures, allowing for the effective release of shear stress. This property enables the thermal recycling and reshaping of the fully crosslinked epoxy–anhydride resin. This strategy not only enhances the functionality of epoxy resins but also broadens their applicability across various thermal and mechanical environments.

## 1. Introduction

Epoxy–anhydride resins are one of most versatile categories of thermosetting polymers characterized by the presence of an epoxy ring [1,2,3], which has wide application in coatings, electronic materials, adhesives, and matrices for fiber-reinforced composites because of their outstanding mechanical properties, high adhesion strength, good heat resistance, and high electrical resistance [4,5,6]. In 2011, Leibler et al. first introduced transesterification into epoxy, allowing it to maintain a crosslinked structure while achieving excellent reprocessing properties [7]. These materials are termed vitrimers, which can enable the rearrangement of network topology through the exchange reactions of dynamic covalent bonds, facilitating the reprocessing of the crosslinked network [8,9]. To enhance the reprocessing performance of epoxy resins, it is often necessary to add transesterification catalysts; otherwise, the network system must possess sufficient hydroxyl groups [10,11,12,13], or the occurrence of the transesterification reaction may require special network structural designs. This, to some extent, may limit their versatility [14,15,16].

Although various types of transesterification catalysts applied to epoxy resins have been developed, it is still essential to create a catalyst that exhibits good compatibility with epoxy resins to facilitate transesterification reactions at elevated temperatures. Currently, commonly used transesterification catalysts include metal salts, organic molecules, and organic salt catalysts [17]. However, metal salt catalysts present a serious problem of heterogeneous dispersion in resins due to their solid state and high melting points, which complicates their presence in epoxy monomers [18,19,20,21]. Similarly, organic salt catalysts also face dispersion issues in solid form, often requiring solvents to assist in dispersion and potentially decomposing during the high-temperature curing process [22,23,24]. Organic catalysts, such as imidazole and the most widely used 1,5,7-triazabicyclo [4.4.0]dec-5-ene (TBD), also need to be heated above the melting temperature to disperse [25,26,27,28,29]. In addition, TBD and its derivates might be susceptible to face thermal decomposition under high temperatures [30,31].

Here, we propose to use a thermally stable liquid transesterification catalyst in epoxy–anhydride resins to solve the compatibility, volatility, and structural instability problems. The catalyst employed is an imidazole derivative, featuring an electron-withdrawing group at the 1-position of the imidazole ring and the steric bulkiness associated with the nitrogen atoms at the 3-position [32]. This liquid-state catalyst exhibits enhanced miscibility with epoxy resins at room temperature. In addition, this catalyst shows high-temperature structural stability, which ensures applications where temperatures exceeding 200 °C are needed. Moreover, the catalyst can effectively promote transesterification above 180 °C, facilitating the heat recycling and reshaping of the obtained epoxy resin.

## 2. Materials and Methods

### 2.1. Materials

Diepoxy bisphenol A-type epoxy D.E.R.331 (DE, Dow Chemical, Midland, MI, USA, epoxy equivalent: 182–192 g/eq), methyl tetrahydrophthalic anhydride (MeTHPA, Meryer, Shanghai, China, 95%), *N*,*N*-dimethylbenzylamine (BDMA, Energy Chemical, Shanghai, China, 97%), bis(2-ethylhexyl) fumarate (2-2EH, J&K, Beijing, China, 98%), 4-phenylimidazole (4PI, J&K, 97%), 1,8-diazabicyclo [5.4.0]undec-7-ene (DBU, J&K, 98%), magnesium sulfate (MgSO_4_, Energy Chemical, 98%), hexane (J&K, AR), ethyl acetate (TGREAG, Beijing, China, GC) triethylamine (TGREAG, 99.5%), and acetonitrile (TGREAG, AR) were used as purchased.

### 2.2. Synthesis of the 1-2EH-4PI

DBU (2.64 g, 17.34 mmol) was added to a mixture of 4PI (5.00 g, 0.04 mol) and 2-2EH (11.81 g, 0.04 mol) in 15 mL of acetonitrile. The concentration of reactants was 59 wt%. The resulting mixture was stirred at room temperature for 0.5 h. After evaporation of the solvent, the residue was dissolved in ethyl acetate, washed three times with saturated saltwater, and dried over MgSO_4_. Purification was carried out using column chromatography on silica gel with a hexane/ethyl acetate (8:1, *v*/*v*) mixture, to which a few drops of triethylamine were added. This process yielded 1-2EH-4PI as a pale brown liquid.

### 2.3. Synthesis of the EP-Epoxy Resin

The epoxy resin containing 1-2EH-4PI (abbreviated as ep-epoxy resin) was synthesized by adding 2.225 g of MTHPA, 2.5 g of epoxy 331, 0.035 g of BDMA, and 0.236 g of catalyst 1-2EH-4PI to a 50 mL round-bottom flask. No solvents were needed. The mixture was preheated at 60 °C and stirred until they were mixed thoroughly. The catalyst 1-2EH-4PI was evenly dispersed, and the reaction mixture showed a light brown color. The mixture was poured into a rectangular silicone mold. Subsequently, the mold was placed in a vacuum oven at 80 °C to be degassed, and then heated for 4 h for pre-curing. After that, the pre-cured sample was heated at 140 °C for 12 h to obtain the epoxy resin sample.

### 2.4. Differential Scanning Calorimetry (DSC) Tests

DSC tests were performed on TA instruments Q2000 (New Castle, DE, USA). The thermal property of 1-2EH-4PI was tested at a rate of 10 °C min^−1^ between 0 °C and 200 °C. As for the thermal property of the ep-epoxy, a pre-treatment cycle from 0 °C to 200 °C was applied to remove its thermal history, which was conducted at a rate of 30 °C min^−1^. The measurement of glass transition temperature (*T*_g_) of ep-epoxy was realized at a scanning rate of 10 °C min^−1^ from −50 °C to 180 °C.

### 2.5. Shear Stress–Relaxation Experiments

Shear stress–relaxation experiments were performed on a TA-ARG2 rheometer using an 8 mm parallel plate geometry. The unorientated LCE sample was equilibrated at a specific temperature for 2 min before applying a constant strain *γ* of 1%, and a normal force of 10 N was applied to ensure good contact.

### 2.6. Fourier Transform Infrared (FTIR) Spectroscopy Test

The FTIR spectra were tested by a PerkinElmer Spectrum 100 FT-IR Spectrometer (Waltham, MA, USA). Wavelengths were scanned from 4000 cm^−1^ to 600 cm^−1^ under a nitrogen atmosphere.

### 2.7. Nuclear Magnetic Resonance (NMR) Spectroscopy Measurements

^1^H and ^13^C NMR spectroscopy measurements were performed in dry NMR tubes on a JEOL JNM-ECA400 (400 MHz) spectrometer (JEOL Co., Ltd., Tokyo, Japan) in DMSO-d_6_ or CDCl_3_ using tetramethylsilane (TMS; d = 0 ppm) as the internal reference. Samples of 10 mg were analyzed and the obtained data were internally referenced to the standard shift of the respective solvent.

### 2.8. Mass Spectrometry

Mass spectrometry was conducted on a MALDI-TOF MS (Shimadzu, Kyoto, Japan).

### 2.9. Thermogravimetric Analysis (TGA)

TGA was used to determine the degradation temperature of samples. The TGA results of the 1-2EH-4PI and the ep-epoxy resin were obtained on TA instruments Q50 under air atmosphere with a heating rate of 10 °C min^−1^ from room temperature to 800 °C.

## 3. Results

### 3.1. Synthesis and the Thermal Stability of 1-2EH-4PI

**Figure 1:**
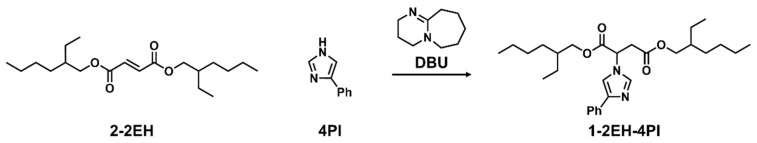
The synthesis of 1-2EH-4PI.

The liquid catalyst was synthesized using an aza-Michael reaction between 2-2EH and 4PI according to the previously reported work (Figure 1) [32]. The 2-2EH was applied as a branched bulky substituted group on the 1-position of the imidazole ring to increase the steric bulkiness (Figures S1–S3).

**Figure 2:**
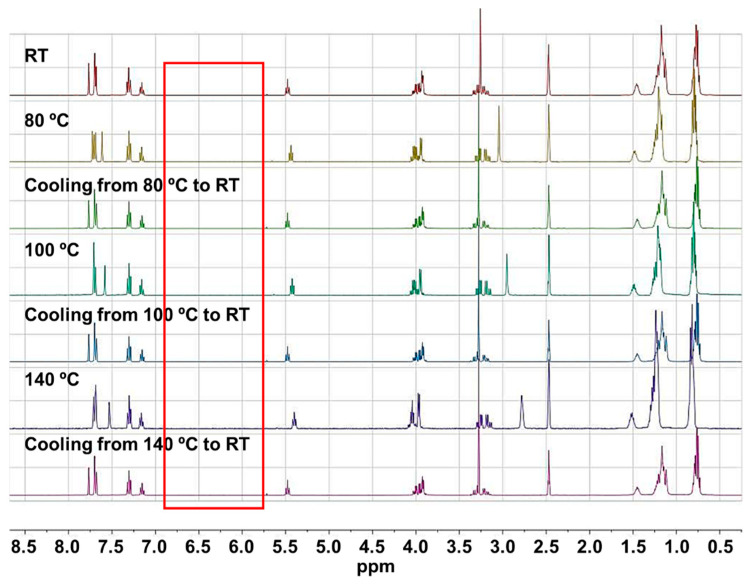
^1^H NMR spectra of 1-2EH-4PI at varied temperatures (400 MHz, DMSO-d6).

The catalyst 1-2EH-4PI demonstrated significant high-temperature stability, with no evidence of retro aza-Michael reaction occurring (Figure 2). ^1^H NMR spectra indicated the absence of olefin protons corresponding to fumarate 2-2EH, which would typically appear around 6.8 ppm when heated to 140 °C. Due to the boiling point of the DMSO-d6, tests at higher temperatures were not conducted.

**Figure 3:**
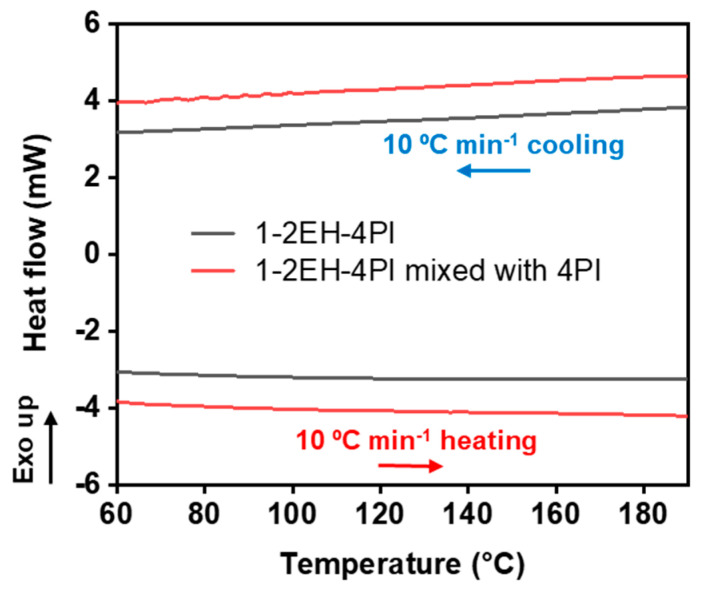
DSC curves of 1-2EH-4PI (the gray line) and the mixture of 1-2EH-4PI and 4PI (the red line).

DSC curves also revealed no distinct peaks during the heating and cooling cycle between 50 and 200 °C, further confirming the thermal stability of the material (Figure 3). Even when the 4PI monomer was mixed with 1-2EH-4PI, there was still no observable retro aza-Michael reaction. Moreover, the TGA results showed that the 1% weight loss temperature was 230.0 °C and the 5% weight loss temperature was 269.7 °C (Figure S4).

**Figure 4:**
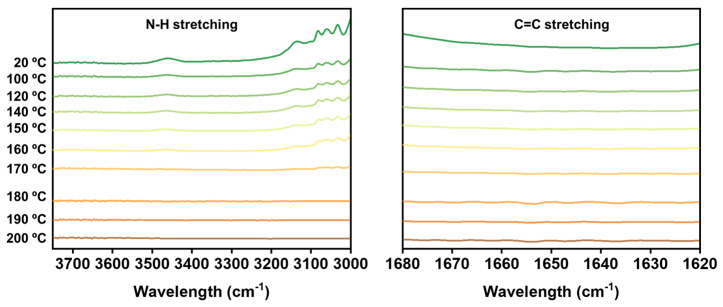
FTIR results of the 1-2EH-4PI at elevated temperatures.

In addition, Fourier transform infrared spectroscopy (FTIR) tests were also conducted to investigate the stability of 1-2RH-4PI (Figure 4). If 1-2EH-4PI undergoes a retro aza-Michael reaction and dissociates at high temperatures, a new N-H stretching vibration peak should appear in the 3500–3300 cm^−1^ range, along with a C=C stretching peak in the 1680–1620 cm^−1^ range during the heating process. However, test results show that no new peaks appear in these two regions as the temperature increases to 200 °C, indicating that the material maintains its structural integrity under the tested thermal conditions.

### 3.2. Network Design of the EP-Epoxy Resin

**Figure 5:**
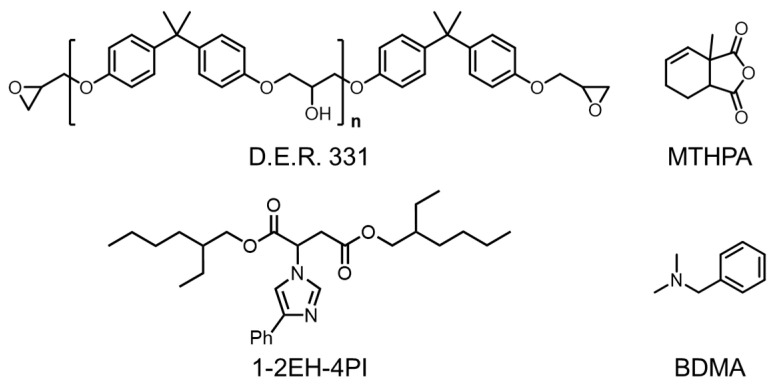
Chemical composition of the ep-epoxy resin.

As Figure 5 shows, the ep-epoxy resin was formulated using D.E.R. 331 epoxy and MeTHPA as the primary components. To facilitate the curing process, 1 wt% BDMA was incorporated into the formulation. In addition, for transesterification purposes, 5 wt% of 1-2EH-4PI was included in the mixture. The DSC results revealed that the glass transition temperature (*T*_g_) of the sample was approximately 110 °C (Figure S5).

### 3.3. Investigation of Transesterification

**Figure 6:**
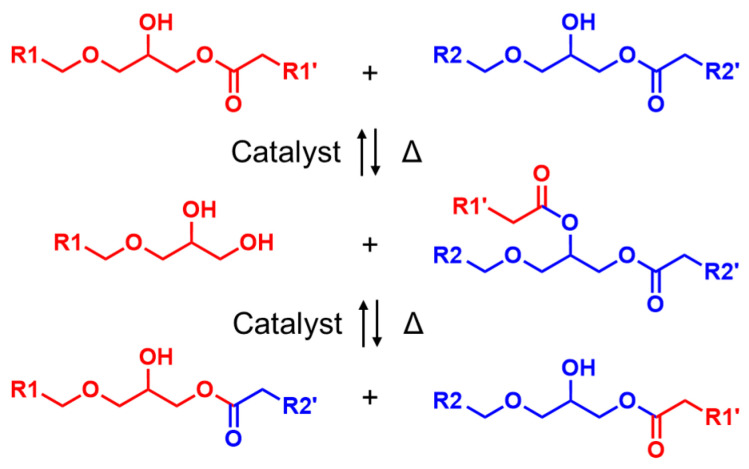
Mechanism of transesterification.

**Figure 7:**
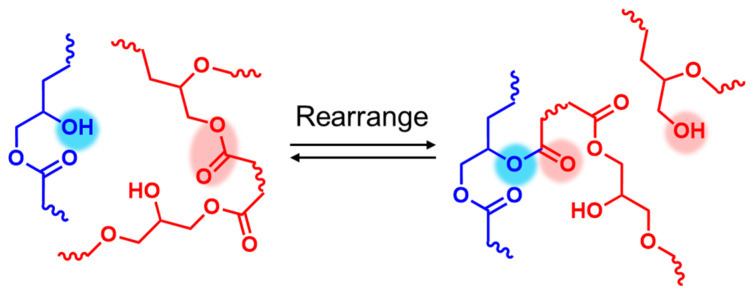
Rearrangement of network topology.

The use of 1-2EH-4PI facilitated transesterification at elevated temperatures (Figure 6). The dynamic ester linkages can break and reform through an associative mechanism, allowing the epoxy resin to maintain its network integrity during reprocessing (Figure 7).

**Figure 8:**
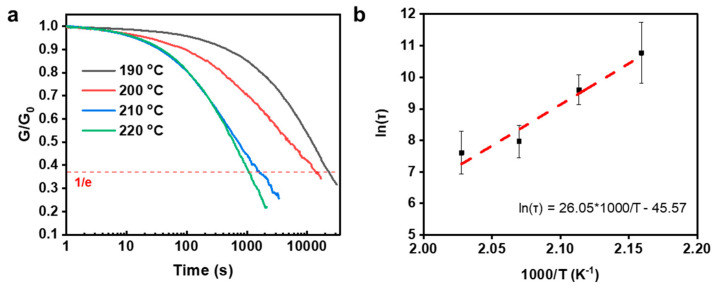
Calculation of *E*_a_ of the ep-epoxy resin. (**a**) Shear stress relaxation tests of the ep-epoxy resin. (**b**) Fitting line of the Arrhenius relationship. * stands for multiplication.

Shear stress relaxation tests were conducted to measure the impact of 1-2EH-4PI on transesterification within the network (Figure 8a). The results showed that the viscosity–temperature relationship of the ep-epoxy resin followed the characteristic Arrhenius relationship. The relationship is defined by the following equation:

(1) $$\tau^{*}=\tau_{0}exp\left(\frac{E_{a}}{RT}\right)$$

τ* is the characteristic relaxation time, which is defined as the time required for the normalized modulus to decrease to e^−1^ (∼37%) from the initial value. The activation energy (*E*_a_) is the Arrhenius activation energy. R is the universal gas constant. *T* is the temperature. *E*_a_ of transesterification in the ep-epoxy resin can be calculated by plotting ln(τ) vs. 1000/T to yield a fitting straight line. The slope is correlated to *E*_a_. Therefore, the *E*a was calculated to be 216.56 kJ mol^−1^ (Figure 8b).

### 3.4. Reprocessability of the EP-Epoxy Resin

**Figure 9:**
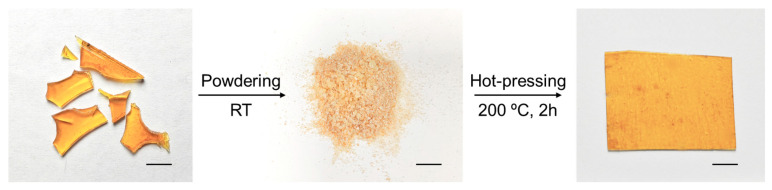
Thermally recyclability of the discarded ep-epoxy sample. Scale bars: 5 mm.

The obtained epoxy can be thermally recycled. As Figure 9 shows, some discarded epoxy samples were pulverized into a powder. The epoxy powder was then heat-pressed at 200 °C under 5 MPa. After 2 h, a uniform sample film was produced, indicating that the catalyst 1-2EH-4PI effectively facilitated the thermal recyclability of the ep-epoxy resin. The original resin and reprocessed resin have similar properties, as shown by the SEM images, IR spectra, TGA curves, DSC curves, and tensile curves (Figures S6–S10).

**Figure 10:**
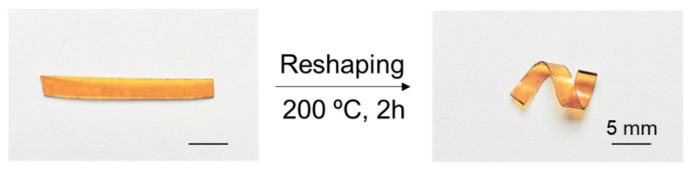
Thermal reshaping of the ep-epoxy sample.

The ep-epoxy resin can also be reshaped upon heating (Figure 10). In this process, the sample was first cut into a flat film and subsequently reshaped into a spiral form. The reshaped sample was then heated at 200 °C for 2 h. Following this treatment, the new shape was permanently retained, demonstrating the material’s capacity for thermal reconfiguration while maintaining structural integrity. This property enhances the epoxy’s versatility for a range of applications.

## 4. Conclusions

In conclusion, this work introduces a thermally stable liquid transesterification catalyst, 1-2EH-4PI, into epoxy–anhydride resins to address the miscibility and stability issues of previous catalysts. This catalyst can be easily dispersed into the epoxy resin components at room temperature while maintaining robust structural stability at elevated temperatures. Furthermore, its high catalytic efficiency enables effective heat reprocessing and reshaping of the epoxy resins at higher temperatures. These advantages make the 1-2EH-4PI contained epoxy–anhydride have extended potential industrial applications in high-temperature servicing conditions such as solid-state insulated transformers, space launchers, and aircraft engines.

In the future, further optimization of the catalyst’s structure could tune its catalytic temperature to broaden its applicability in various thermal processing scenarios. For example, investigating alternatives of 2-2EH and 4PI can yield various series of liquid-state catalyst derivates to regulate the catalyzing temperature for different application scenarios. In addition, using TBD to replace 4PI may lead to improved performance, enabling more versatile uses of epoxy resins in advanced materials and applications.

## Data Availability

The original contributions presented in the study are included in the article, further inquiries can be directed to the corresponding author.

## Supplementary Materials

The following supporting information can be downloaded at: https://www.mdpi.com/article/10.3390/polym16223216/s1, Figure S1: Mass spectrometry results of the ep-epoxy; Figure S2: The chemical shifts in characteristic peaks of 1-2EH-4PI. ^1^H NMR (400 MHz, CDCl_3_); Figure S3: The chemical shifts in characteristic peaks of 1-2EH-4PI. ^13^C NMR (400 MHz, CDCl_3_); Figure S4: TGA result of the catalyst 1-2EH-4PI; Figure S5: DSC curve of the ep-epoxy; Figure S6: SEM images of the ep-epoxy resin before and after reprocessing; Figure S7: IR spectra of the ep-epoxy resin before and after reprocessing; Figure S8: TGA curves of the ep-epoxy resin before and after reprocessing; Figure S9: DSC curves of the ep-epoxy resin before and after reprocessing; Figure S10: Tensile curves of the ep-epoxy resin before and after reprocessing.
