# Supplementary material for: Reprocessable Epoxy–Anhydride Resin Enabled by a Thermally Stable Liquid Transesterification Catalyst
Source: Polymers (Basel). 2024 Nov 20;16(22):3216. doi: 10.3390/polym16223216 (PMC11598466; doi:10.3390/polym16223216)
Supplement: Supplementary file 1 [file polymers-16-03216-s001.zip › polymers-3296301-supplementary.pdf]

Supplementary Materials

Reprocessable Epoxy–Anhydride Resin Enabled by a Thermal-Stable Liquid Transesterification Catalyst

Huan Liang[1], Wendi tian[2], Hongtu Xu[1], Yuzhen Ge[1], Yang Yang[3], Enjian He[1], Zhijun Yang[1], Yixuan Wang[1], Shuhan Zhang[1], Guoli Wang[4], Qiulin Chen[4], Yen Wei[1,*] and Yan Ji[1,*]

1 Key Laboratory of Bioorganic Phosphorus Chemistry & Chemical Biology (Ministry of Education), Department of Chemistry, Tsinghua University, Beijing 100084, China.

2 School of Materials Science and Engineering, University of Science and Technology Beijing, Beijing 100083, China.

3 Institute of Nuclear and New Energy Technology, Tsinghua University, Beijing 100084, China

4 Electric Power Research Institute, China Southern Power Grid Co. Ltd, Guangzhou 510623, China.

* Correspondence: weiyen@tsinghua.edu.cn; jiyan@mail.tsinghua.edu.cn.

Table of Contents

Figure S1. Mass spectrometry results of the ep-epoxy..2

Figure S2. The chemical shifts of characteristic peaks of 1-2EH-4PI. ^{1}H NMR (400 MHz, $CDCl_3$). 3

Figure S3. The chemical shifts of characteristic peaks of 1-2EH-4PI. ^{13}C NMR (400 MHz, $CDCl_3$). 4

Figure S4. TGA result of the catalyst 1-2EH-4PI. 4

Figure S5. DSC curve of the ep-epoxy...5

Figure S6. SEM images of the ep-epoxy resin before and after reprocessing. 5

Figure S7. IR spectra of the ep-epoxy resin before and after reprocessing. 6

Figure S8. TGA curves of the ep-epoxy resin before and after reprocessing................6

Figure S9. DSC curves of the ep-epoxy resin before and after reprocessing. 7

Figure S10. Tensile curves of the ep-epoxy resin before and after reprocessing. 7

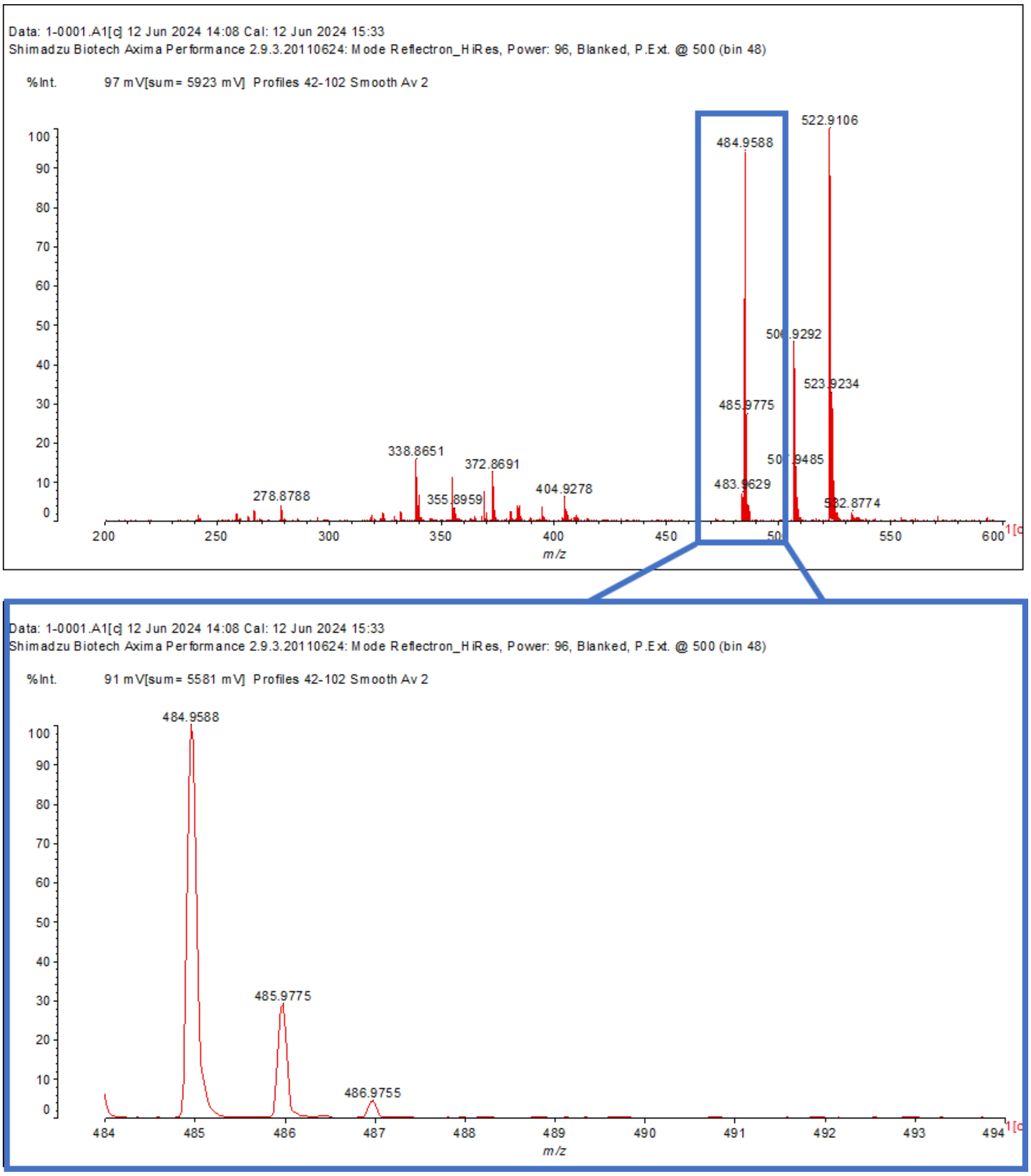

Figure S1. Mass spectrometry results of the ep-epoxy.

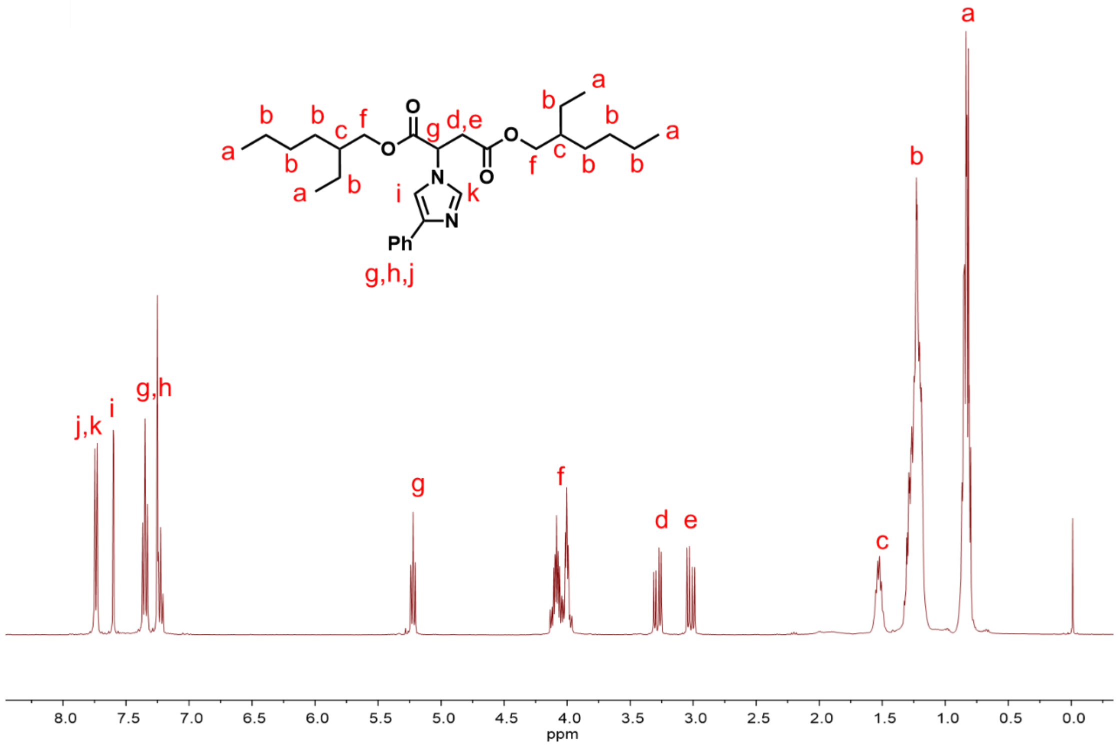

Figure S2. The chemical shifts of characteristic peaks of 1-2EH-4PI. ^{1}H NMR (400 MHz, $CDCl_3$).

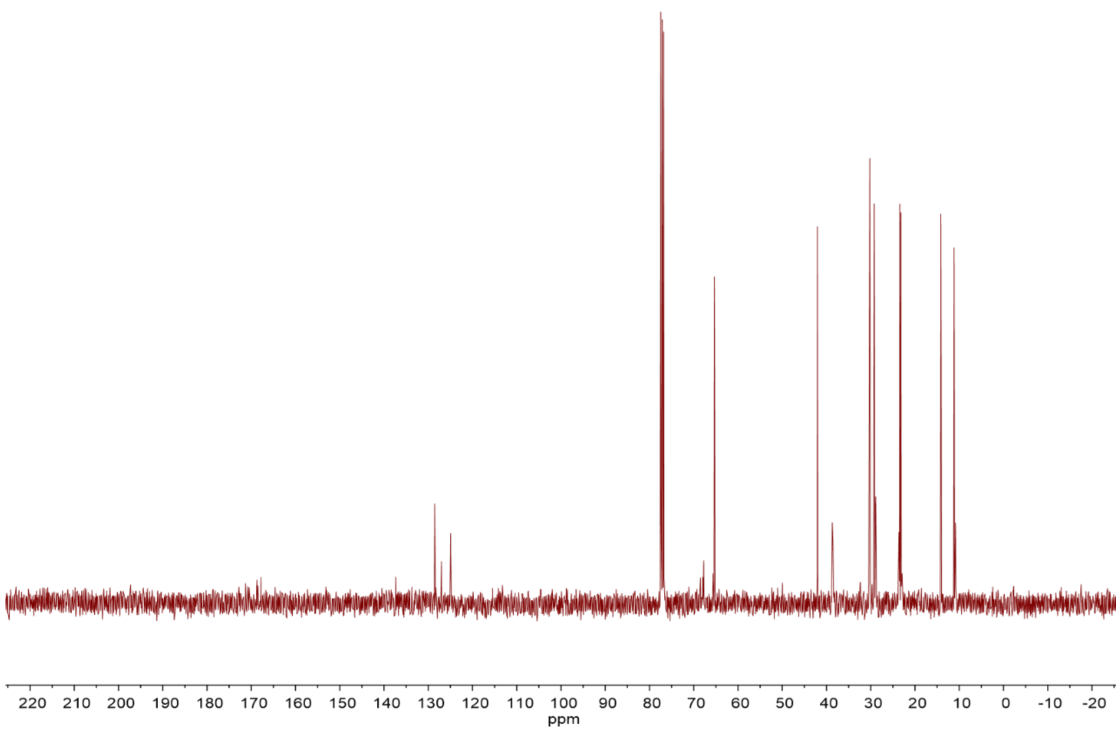

Figure S3. The chemical shifts of characteristic peaks of 1-2EH-4PI. ^{13}C NMR (400 MHz, $CDCl_3$).

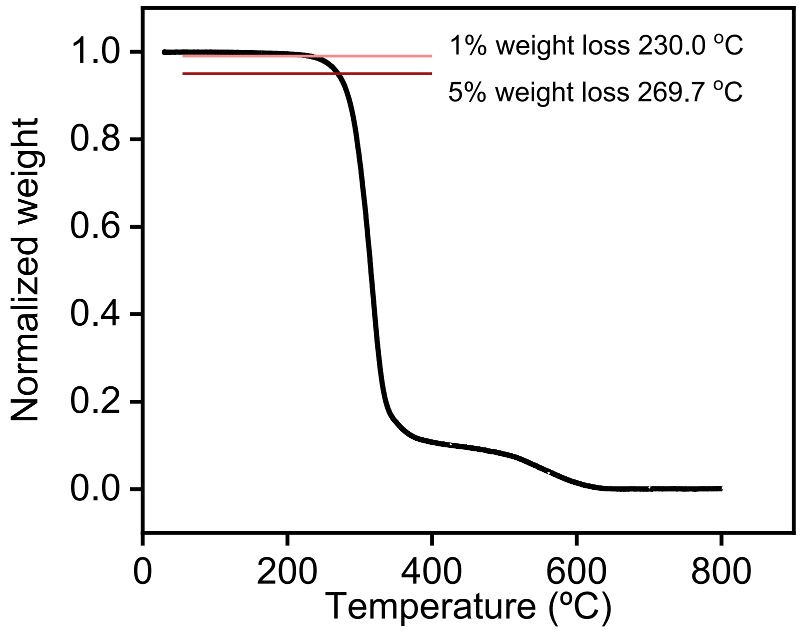

Figure S4. TGA result of the catalyst 1-2EH-4PI.

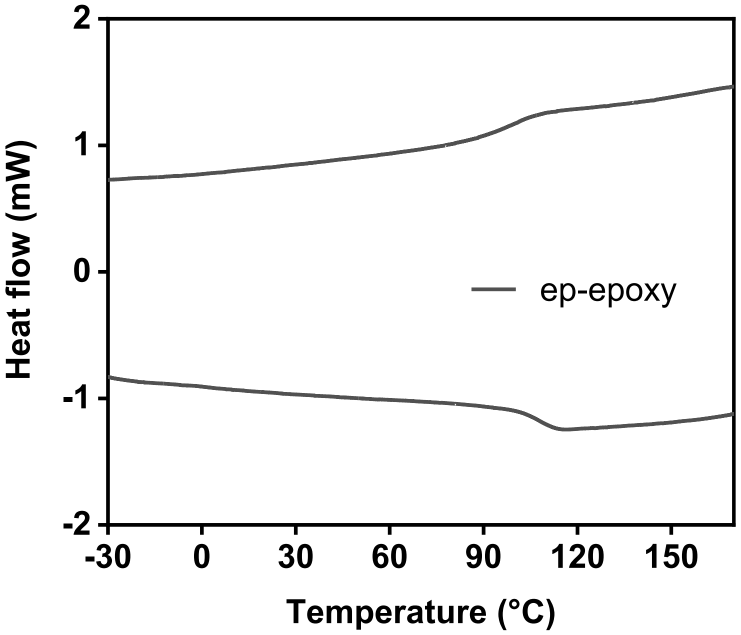

Figure S5. DSC curve of the ep-epoxy.

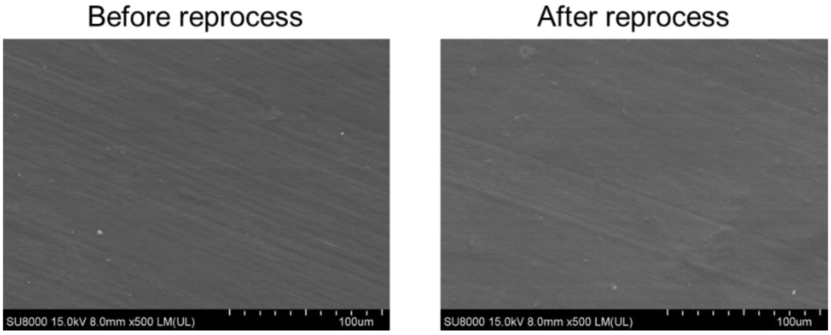

Figure S6. SEM images of the ep-epoxy resin before and after reprocessing. The results showed the appearance of samples before and after reprocessing were similar, indicating the sample was successfully thermal recycled.

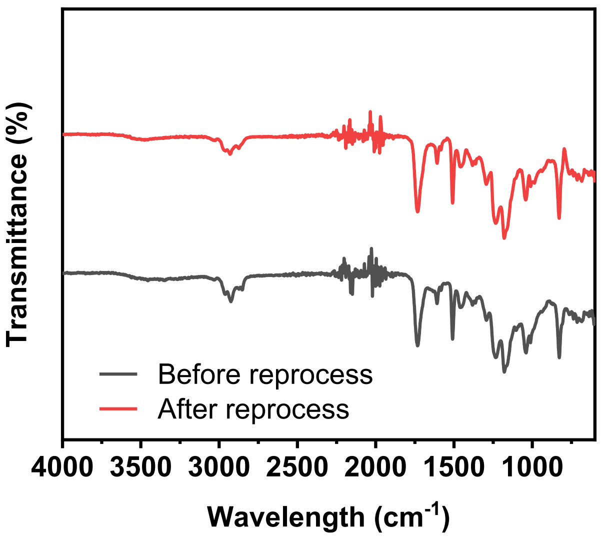

Figure S7. IR spectra of the ep-epoxy resin before and after reprocessing.

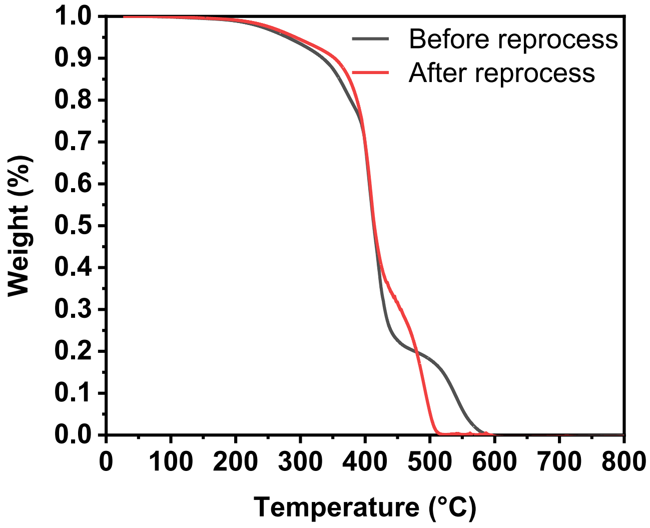

Figure S8. TGA curves of the ep-epoxy resin before and after reprocessing. The curves showed similar 1% weight loss of samples before and after reprocessing (189 ºC and 196ºC, respectively).

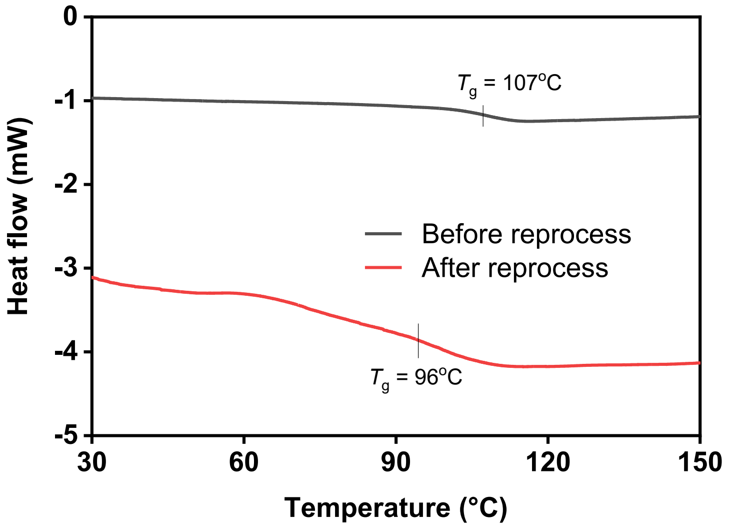

Figure S9. DSC curves of the ep-epoxy resin before and after reprocessing. The curves showed the glass transition temperature of samples before and after reprocessing were similar (107 ºC and 96 ºC, respectively).

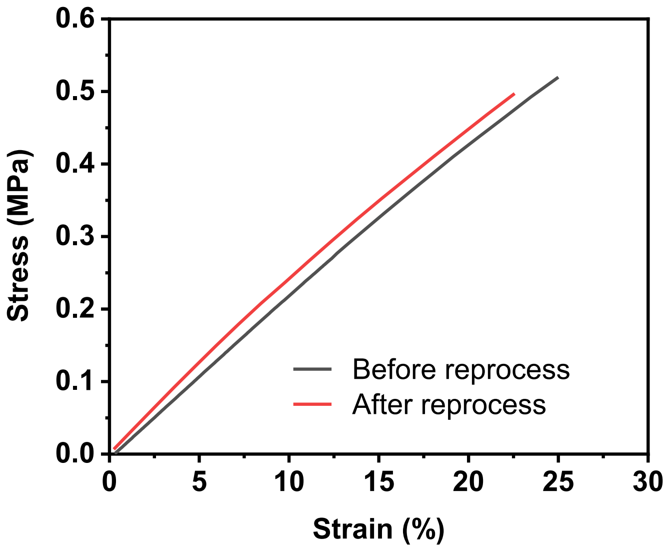

Figure S10. Tensile curves of the ep-epoxy resin before and after reprocessing. This test was conduct at 140ºC since the sample was too brittle below T_g, making it difficult to test.
